# Live-cell imaging in the deep learning era

**DOI:** 10.1016/j.ceb.2023.102271

**Published:** 2023-10-27

**Authors:** Joanna W. Pylvänäinen, Estibaliz Gómez-de-Mariscal, Ricardo Henriques, Guillaume Jacquemet

**Affiliations:** 1 Faculty of Science and Engineering, Cell Biology, Åbo Akademi, University, 20520 Turku, Finland; 2 Instituto Gulbenkian de Ciência, Oeiras 2780-156, Portugal; 3 University College London, London WC1E 6BT, United Kingdom; 4 Turku Bioscience Centre, University of Turku and Åbo Akademi University, 20520, Turku, Finland; 5 InFLAMES Research Flagship Center, University of Turku and Åbo Akademi University, 20520, Turku, Finland; 6 Turku Bioimaging, University of Turku and Åbo Akademi University, FI-20520 Turku, Finland

## Abstract

Live imaging is a powerful tool, enabling scientists to observe living organisms in real time. In particular, when combined with fluorescence microscopy, live imaging allows the monitoring of cellular components with high sensitivity and specificity. Yet, due to critical challenges (i.e., drift, phototoxicity, dataset size), implementing live imaging and analyzing the resulting datasets is rarely straightforward. Over the past years, the development of bioimage analysis tools, including deep learning, is changing how we perform live imaging. Here we briefly cover important computational methods aiding live imaging and carrying out key tasks such as drift correction, denoising, super-resolution imaging, artificial labeling, tracking, and time series analysis. We also cover recent advances in self-driving microscopy.

## Introduction

**Figure 1:**
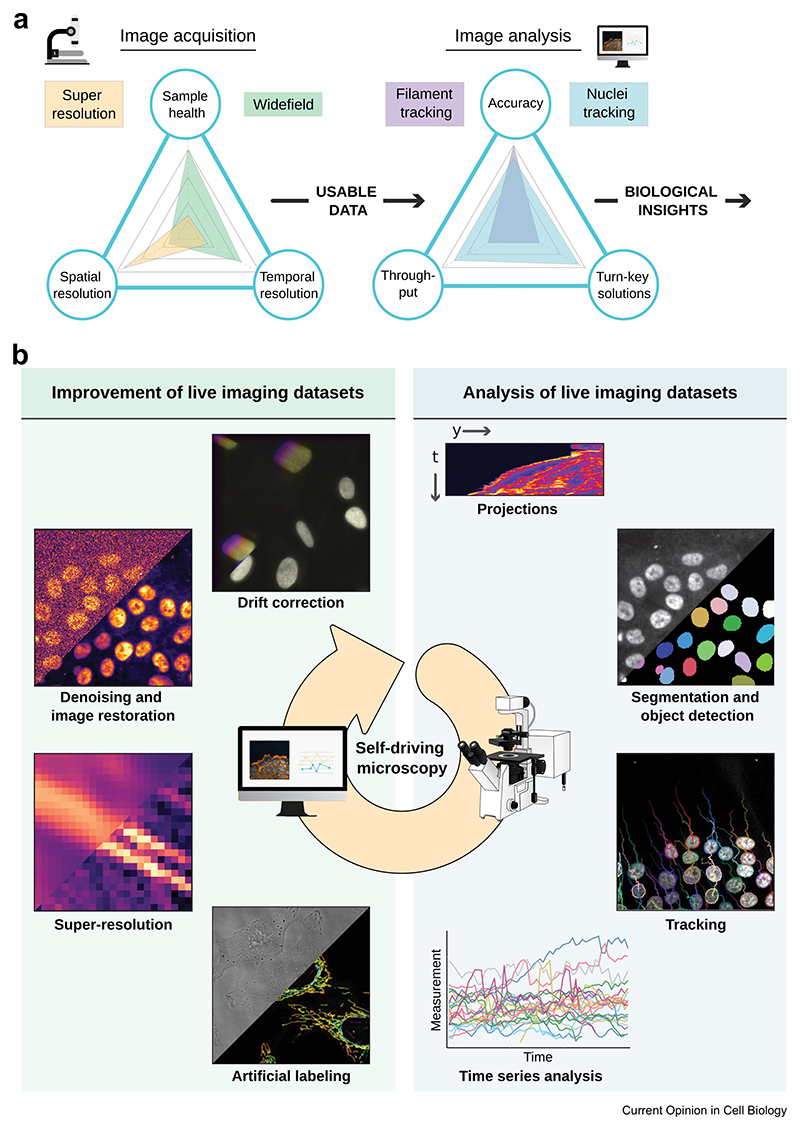
Live-cell imaging main challenges and computational solutions. (**a**) Live fluorescence imaging presents unique challenges that require a careful balance between managing light sensitivity and ensuring optimal spatial, temporal, and spectral resolution to observe intended biological phenomena accurately. Upon data acquisition, researchers need to select the most effective methods to derive biological insights from their video, with strategies spanning from manual analysis to turn-key solutions or custom-developed analysis pipelines. Each approach has strengths and limitations, particularly throughput, speed and accuracy. This figure, illustrated as spider plots, underscores the need for trade-offs in acquiring and analyzing live imaging data. (**b**) Computational tools designed to handle live cell imaging datasets can be primarily divided into two categories: (i) tools that improve live cell imaging data and mitigate phototoxicity and (ii) tools that facilitate data extraction and analysis. The former category includes methods for drift correction, denoising, resolution enhancement, and artificial labeling. The latter encompasses segmentation, object detection, and tracking tools, followed by time series analysis. Integrating these tools into microscope acquisition software to autonomously control microscope acquisition parameters paves the way for self-driving microscopes. The tool categories are displayed in no particular order, as their use depends on the datasets and needs. The central arrow illustrates that self-driving microscopes can dynamically utilize these approaches to control microscope acquisition parameters.

Live imaging helps us understand life’s complexity by recording how tissues, cells, and molecules behave over time. Yet, implementing live imaging and analyzing microscopy videos remain challenging (Figure 1a). Firstly, live imaging is frequently susceptible to drift, leading to an unwanted sample displacement over time. Secondly, when using fluorescence microscopy, balancing between imaging frequency, resolution, and specimen health is critical and challenging [1]. Finally, live imaging experiments tend to generate an avalanche of data that can be hard to extract and analyze (Figure 1a).

To mitigate some of these issues, there is ongoing work on hardware improvement. For example, gentler illumination strategies and more sensitive detectors can reduce phototoxicity [2,3]. However, hardware improvements are only part of the solution. Increasingly, powerful software advancements enhance microscopy, providing us with more information from our samples (e.g., Refs. [4–6]). Over the past few years, significant strides have been made in data processing tools, broadly categorized into 1) tools aiming at improving live imaging datasets and 2) tools aiming at extracting quantitative information from live imaging datasets (Figure 1b). Many of these tools now operate through deep learning (DL), a subfield of artificial intelligence that can autonomously identify relevant image features to carry out specific tasks. This review highlights concepts and recent tools useful for researchers interested in live imaging. The tools and articles highlighted are selected based on our experience working with live imaging data and our enthusiasm for this rapidly evolving field. This review is not exhaustive, instead we aim to offer a concise overview of available tools to inspire and empower users.

## Deep learning and video analysis

**Figure 2:**
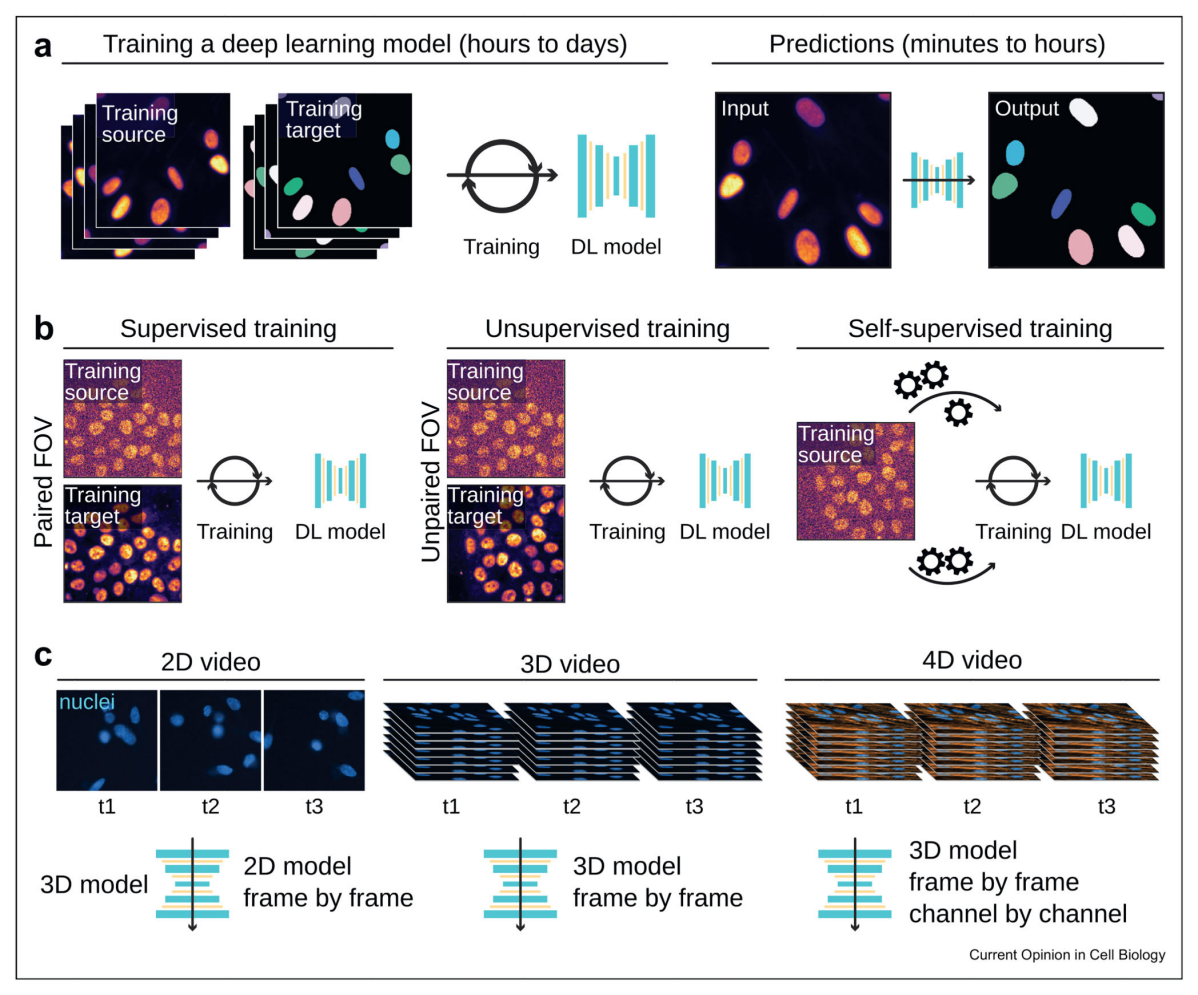
Deep learning and video analysis. (**a**) **The DL pipeline.** A DL model must first be trained using a training dataset. This step is generally time-consuming and takes hours to weeks, depending on the size of the training dataset. Once trained, a model can be directly applied to other images and generate predictions. This second step is generally much faster (seconds to minutes). (**b**) **Type of training datasets.** In a supervised training fashion, a collection of representative input images, each coupled with their anticipated results (i.e., the ground truth), is given to the DNN. Here, the training dataset includes matching pairs of noisy and high signal-to-noise ratio images. Alternate training methods include unsupervised training, where the model is trained with inputs and outputs not necessarily from the same field of view, and self-supervised training, where paired datasets are generated solely from the input images. (**c**) **DL and data dimensions.** Live cell imaging datasets can have multiple dimensions. Given that DL tools for bioimage analysis are typically designed to handle up to three dimensions, applying these tools to video processing necessitates varied strategies, contingent on the number of dimensions present in the data for processing. Here a 2D model represent a model capable to process 2D data. A 3D model is capable to process 3D data. The microscopy images displayed for all panels are breast cancer cells labeled with silicon rhodamine DNA to visualize the nuclei and imaged using a spinning disk confocal microscope.

DL is revolutionizing our ability to analyze microscopy images (see Refs. [7,8] for in-depth reviews). When using DL, a multi-layer artificial neural network, also known as a Deep Neural Network (DNN), is first trained on a dataset to create a “model” capable of executing a specific bioimage analysis task (Figure 2a). Once trained, the model can then be used on similar images. Because of this, the training step is essential as it dictates the performance and specificity of the DNN [9].

When selecting a DL method for processing live imaging data, users must consider the type of training data the chosen approach requires, along with the dimensionality of their data. Typically, DL methods are trained in a supervised manner and rely on paired sets of images. However, alternate strategies to deal with unpaired datasets exist, such as unsupervised and self-supervised training (Figure 2b). Depending on the data available, these methods may offer additional flexibility.

A key characteristic of microscopy videos is the inherent consistency of information across sequential frames. Exploiting this temporal consistency can significantly enhance the precision of data analysis. DL algorithms, adept at handling multi-dimensional data, are particularly effective in analyzing 3D microscopy datasets.

However, the current landscape of DL methods for bioimage analysis is focused on 2D and 3D volumetric datasets. As a result, analyzing 2D, 3D, and 4D videos using DL is often performed frame by frame, which overlooks the time consistency in the data. 3D volumetric approaches can be used to process 2D videos, but this might not fully harness the potential of the data and is generally suboptimal in terms of memory (Figure 2c).

## Drift and bleach correction

**Figure 3:**
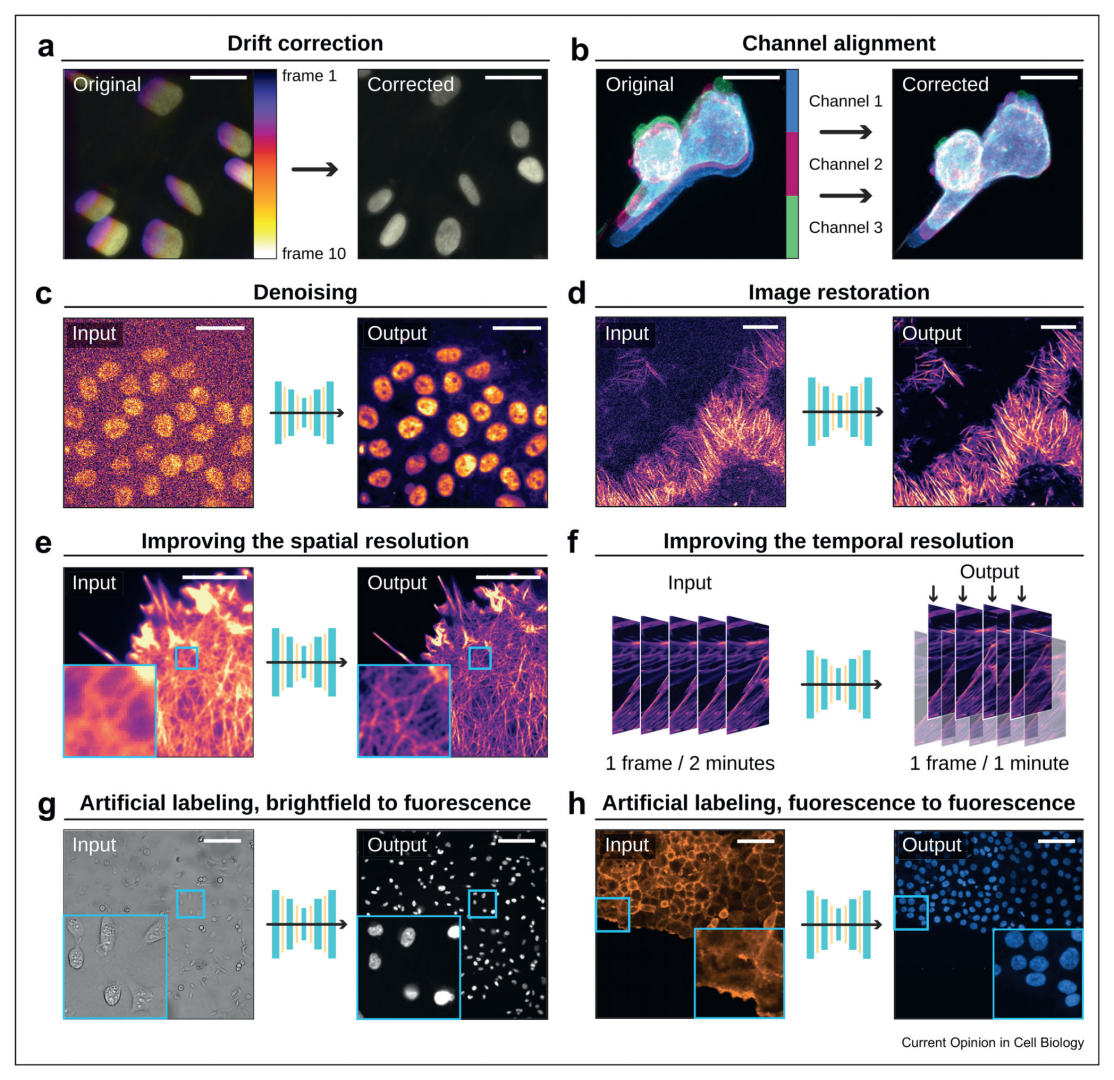
Example of computational tools that can improve live cell imaging movies. This figure illustrates the power and versatility of computational tools in enhancing the quality, resolution, and content of various types of microscopy images. (**a**) Time projection of drifting live images of nuclei, captured by a widefield microscope, corrected using Fast4DReg [10]. The color gradient, transitioning from purple (first frame) to white (last frame), denotes the temporal progression—scale bar: 50 μm. (**b**) A cancer cell in the mouse lung vasculature, in motion and imaged via an Airyscan confocal microscope, is displayed through a maximum-intensity projection. Channel misalignment has been corrected using Fast4DReg [10]—scale bar: 10 μm. (**c**) Noisy images of nuclei, acquired using a spinning disk confocal microscope, were denoised using a CARE 2D model ([17], as described in Ref. [9])—scale bar: 50 μm. (**d**) Breast cancer cells labeled with lifeact-RFP were imaged live using 3D SIM. Images were restored using a CARE 3D model ([17], as described in Ref. [19])—scale bar: 5 μm. (**e**) Cells labeled with Lifeact were imaged using a widefield microscope [45]. The increased image resolution was achieved using the DFCAN deep learning network (as described in Ref. [33])—scale bar: 5 μm. (**f**) This illustration showcases how a DL network like CAFI can enrich the temporal resolution of a live cell imaging dataset through smart interpolations [39]. (**g**) Brightfield microscopy was used to image migrating breast cancer cells, and the nuclei image was digitally generated from the brightfield image using a Pix2pix model [46]—scale bar: 100 μm. (**h**) Breast cancer cells labeled with lifeact-RFP were imaged using a spinning disk confocal. The nuclei image was digitally generated from the lifeact image using a Pix2pix model ([46], as described in Ref. [19])—scale bar: 100 μm.

Microscopy videos must often be corrected to ensure consistency across time frames. This can include removing unwanted drift and upholding image quality throughout the video. While DL algorithms can perform these tasks, they are generally not used for this purpose due to their slow speed or the lack of appropriate training datasets. Drift correction accounts for unwanted shifts in the position of the specimen over time, ensuring consistent frame and channel alignment (Figure 3a and b). For this purpose, we routinely use Fast4DReg [10] and Correct 3D drift [11]. Bleach correction addresses the signal loss occurring when specimens are exposed to too much light over prolonged periods or to uneven illumination. To correct our movies, we routinely use the Bleach correction ImageJ plugin (also available in Napari) [12,13].

## Denoising and restoring live imaging data

Fluorescent live imaging necessitates low concentrations of fluorescent labels and minimal laser power to prevent the disruption of biological processes and ensure the sample’s health. This often leads to the acquisition of noisy images. DL has been successfully applied to remove this noise while preserving the useful signal, thereby facilitating the extraction of meaningful biological information from the imaging data (i.e., [14,15]). DL-based denoising algorithms can be broadly categorized into two groups based on the required training datasets: (i) supervised and (ii) self-supervised (for deeper review, see Ref. [16].

Supervised DL algorithms, such as CARE [17] and 3D-RCAN [18], necessitate paired high- and low-quality images for training. Remarkably, these tools often extend beyond denoising tasks; they serve as comprehensive image restoration algorithms capable of enhancing resolution and eliminating image artifacts, provided they are trained with an appropriate dataset (Figure 3c and d) [9]. These algorithms are changing how live imaging experiments are planned. Indeed, several strategies can be used to generate training datasets to denoise live imaging data, such as using fixed samples [19,20], artificially generating noisy data [21], or collecting live data before or during the timelapse acquisition [22].

Self-supervised algorithms such as Noise2Void [23] allow the training of denoising models directly from noisy images. These algorithms generally assume that the noise is independent of the pixel location (e.g., Gaussian or Poisson noise). If the assumption is met, these approaches can yield results comparable to supervised training without needing a paired training dataset. However, these algorithms may not always be suitable if the noise spatial-independence assumption is unmet (e.g., structured noise) [24]).

Current state-of-the-art denoising methods integrate the knowledge about the image formation process into the learning process, which results in impressive results (i.e., [25,26]). User interested in denoising may consider Aydin witch provides a number of self-supervised, auto-tuned, and unsupervised image denoising algorithms [27]. Of note, it is generally advised to avoid quantifying absolute pixel intensities after DL-based denoising, as DL processing may introduce non-linear changes to the data.

## Improving the spatiotemporal resolution of live imaging data

Live cell imaging aims to capture rich spatiotemporal information while minimizing sample damage. However, light microscopy’s ~250 nm diffraction limit hinders detailed visualization. While various super-resolution strategies exist [28], they rarely suit extended live imaging due to their high laser power requirements. Several analytical methods have demonstrated the capacity to enhance live imaging resolution. Examples of recent non-DL algorithms that improve the resolution of live imaging data include eSRRF [29], SACD [30], and BF-SIM [31]. Super-resolution DL algorithms for live imaging fall into two categories. Algorithms such as SFSRM or DFCAN can super-pixelate an image and predict missing details (Figure 3e) [21,32,33]. Other DL algorithms can aid the post-processing required by most super-resolution microscopy techniques, including SIM [26,34,35] and single-molecule localization microscopy (SMLM) [36,37].

DL-based algorithms can also be used to recover missing temporal information via smart interpolation. For instance DBlink aid faster live SMLM by performing spatiotemporal interpolation [38]. As another example, CAFI can predict intermediary images post-acquisition, enhancing temporal resolution [39] (Figure 3f).

## Artificial labeling

Artificial labeling is a computational technique that utilizes DL to predict staining based on other microscopy images [40,41]. For instance, artificial labeling can predict a nucleus staining from brightfield or F-actin images (Figure 3g and h). The predicted staining can assist downstream analysis, such as segmentation and tracking [19,20,42]. Artificial labeling is especially beneficial for live imaging as it allows for staining recovery without explicit imaging, thereby improving acquisition speed, multiplexing, and reducing phototoxicity. When combined with live brightfield, phase, or digital holographic imaging, artificial labeling offers a non-invasive, non-destructive approach for comprehensive cellular structure visualization [43,44].

## Segmentation and tracking

**Figure 4:**
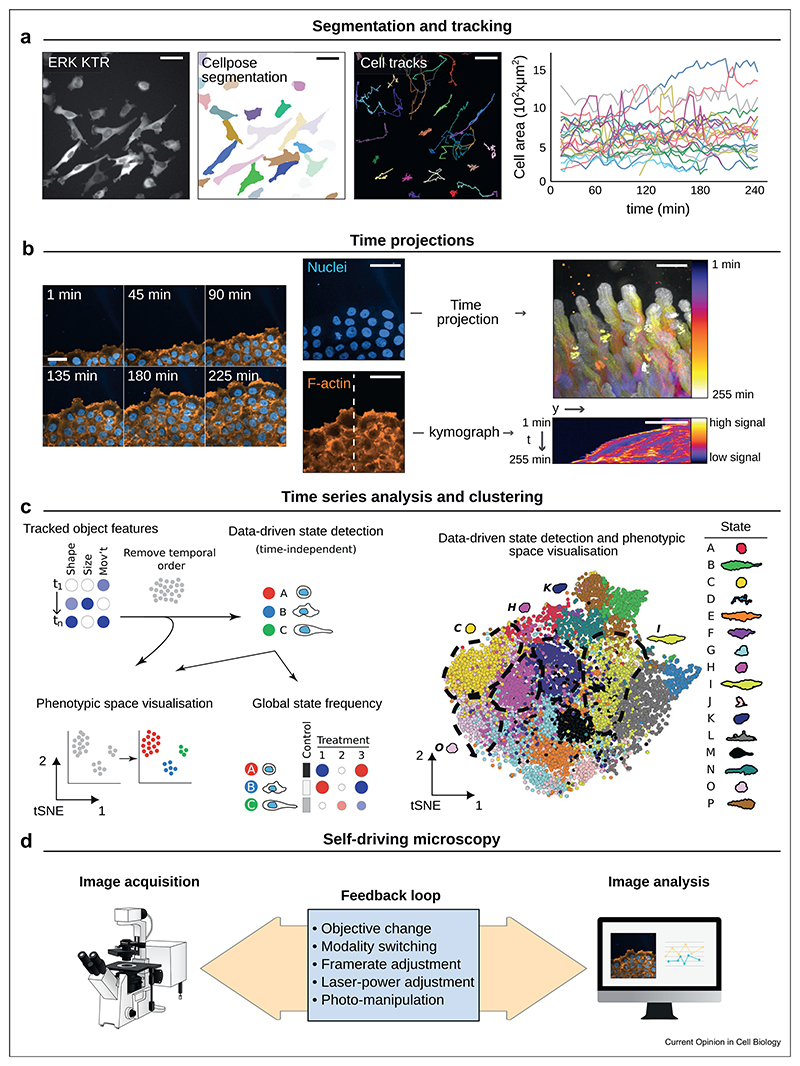
Extracting temporal information from live imaging data. (**a**) Widefield fluorescence microscopy was used to image breast cancer cells expressing a GFP-tagged ERK-reporter (dataset described in Ref. [48]). The cytoplasm was segmented using a custom CellPose model [83], and cell movements were tracked with CellPose in TrackMate [48]. Changes in cell area over time were plotted using PlotTwist [70]—scale bar: 50 μm. (**b**) Lifeact-RFP-expressing cancer cells were recorded using a spinning disk confocal microscope. Dynamic changes are visualized in a single image using a time projection (purple to white) and a kymograph along a defined line—scale bar: 50 μm. (**c**) Cancer cell spheroids were imaged at low resolution using an incubator microscope. After segmentation and tracking, the phenotypic state classification of the spheroids, as well as the visualization of the phenotypic space, was enabled by a data-driven time-series analysis focusing on cell shape, size, and movement (figure panel adapted from Ref. [66], only the font size and image sizes were changed in respect to the original figure). (**d**) Self-driving microscopy provides real-time feedback during image acquisition. Analyzed on the fly, the acquired data enables adjusting microscope settings and acquisition parameters, optimizing data collection.

One key strategy to extract biological information from videos is tracking, which involves following objects of interest over time to quantify their behaviors. Tracking is typically a two-step process: object detection at each time point and tracking formation via detection linking (Figure 4a). Tracking accuracy often relies on successful object recognition, where segmentation methods employing machine learning and DL algorithms have demonstrated proficiency for various bioimages (for review, see Ref. [47]). Because of this, DL segmentation tools are now integrated into tracking platforms, such as TrackMate [48], Cell-ACDC [49], DeepTree [50], and ELEPHANT [51]. These tools cater to different needs based on the nature of the data, required features, and the user’s preferred computational platform. For instance, ELEPHANT aims at tracking objects within large 4D movies. TrackMate, integrated in Fiji [52], is feature-packed and allows, for instance, to follow morphological and intensity changes of the tracked object over time. DL algorithms can also be used for the object linking step [53]. Despite the growing prevalence of DL-based strategies in tracking, cleverly crafted classical algorithms remain state-of-the-art for certain uses, such as the segmentation and tracking of mitochondria [54]. Finally, an integral aspect of automated tracking is verifying the performance of the chosen method for a specific dataset, for which several metrics have been developed to score tracking quality [55–57]. These metrics can also guide the optimization of tracking parameters, ensuring the most accurate and useful data extraction from live imaging data [48].

Yet tracking is not always necessary. Segmentation alone can detect events within video data and yield valuable biological insights (i.e., [58]). While most DL-based segmentation methods for video microscopy are supervised, which requires the creation of a manually labeled training dataset, self-supervised methods also exist. One notable example is Time Arrow Prediction [59], designed to detect time-asymmetric biological processes such as cell division from microscopy videos.

In some conditions, tracking is insufficient. For instance, when studying changes within an object over time. One solution is to use an analysis window strategy, which divides the object into distinct areas for individual assessment [60]. However, this method faces challenges when the tracked objects undergo large deformations during the video (such as shape changes during cell migration) [61]. In this case, nonlinear image registration can be used to align the object outline and interior in each frame, facilitating the spatiotemporal analysis of processes within the object [61].

## Reducing the complexity of live imaging data via projections

Quantitative analysis of multi-dimensional live imaging datasets can be complex. It can be greatly simplified by reducing the video dimensions using projections (such as time projection, Figure 4b) or creating spatiotemporal maps (such as kymographs, Figure 4b), which capture dynamic changes in single images. DL algorithms such as the 4SM model and KymoButler can automate creating and analyzing spatiotemporal maps in large datasets [62,63]. Projections can also be applied to complex datasets, such as light-sheet movies of cancer cells migrating in 3D. For instance, u-Unwrap3D can remap arbitrarily complex 3D cell surfaces into equivalent lower-dimensional representations. This surface-guided projection strategy allows the tracking of segmented surface motifs and associated fluorescent signals in 2D [64].

## Time series analysis

Once numbers are extracted from the video, additional steps often come into play for meaningful analysis and comparison, especially when a simple time series average is insufficient. For instance, time series normalization becomes crucial when following intensity changes over time in single cells. As another example, Granger-causal inference can be used to compare time series and infer cause–effect relations between fluctuating protein intensity recordings [65]. When dealing with high-dimensionality data, clustering, principal components, and t-SNE analyses can significantly assist in the unbiased discovery of rare phenotypes (Figure 4c, [66–68]). Recent advancements include tools like CellPhe and Traject3D, designed to automate cell phenotyping across different imaging modalities [66,67]. In this context, DL algorithms can potentially enhance time series analysis even further [69].

When analyzing time series, online tools like PlotTwist [70] offer a user-friendly platform for straightforward needs. Multiple Python and R toolboxes such as sktime [71] are available for more complex analyses. These packages provide a wide range of methods for time series analysis. Regardless of the chosen approach, quality control is fundamental for time series analysis to ensure result reproducibility, which often relies on standardized procedures combined with batch correction [72].

## Self-driving microscopy

By combining on-the-fly image analysis with automated microscope control, self-driving microscopy software are revolutionizing how we perform live cell imaging experiments [73–76]. For instance, it allows for effortless transitions from low to high-magnification imaging during time-lapse acquisition [74] or modifying imaging rates on the fly, capturing biological events in remarkable detail [76]. The technology also enables modality switching, such as brightfield to fluorescence or wide-field to SR [73,74], presenting unmatched adaptability in live cell imaging. Another significant feature is the ability to control optogenetic stimulation autonomously [75]. The focus on user-defined pertinent events mitigates phototoxicity and photobleaching, safeguarding sample health while optimizing efficiency by reducing unnecessary imaging. Furthermore, it can capture elusive, transient events that could be overlooked by traditional methods, thereby heightening the efficacy of live cell imaging experiments (Figure 4d).

As a burgeoning field, self-driving microscopy holds considerable potential, particularly when coupled with DL’s capacity to leverage imaging data and our ability to execute complex computations in real time. The cornerstone of self-driving microscopes lies in open-source microscopy control software, which enables adaptive control schemes and event detection. Pioneering platforms such as Micro-Manager [77–79], Pycro-Manager [80] or AutoScanJ [81] are at the fore-front, driving these technological advancements and redefining the landscape of live imaging.

## Choosing an image analysis tool

In the rapidly evolving landscape of image analysis tools [82], the choice of approach is strongly influenced by the specific sample being imaged. With a myriad of DL networks, models, and software available, there isn’t a universally optimal tool; instead, the selection depends on the sample imaged, the type of data collected, and the data that needs to be extracted from the video. Tool selection is also influenced by the user’s familiarity with different interfaces and proficiency in coding languages.

Training DL models generally demands significant computational resources and often necessitates coding and computational proficiency. Several tools, such as ZeroCostDL4Mic, Cellpose 2.0, or DeepCell Kiosk, have made DL training and deployment for bioimage analysis more accessible [19,83–85]. In addition, ongoing initiatives facilitate sharing and re-using trained DL models by creating model zoos [83,84,86–89]. While DL approaches generally outperform traditional image processing techniques, it is essential to remember that the latter may be more appropriate or faster to implement.

When using DL, users should craft their training dataset carefully and, in particular, ensure that their sample heterogeneity is well represented in the training data-set. We also recommend that users take the time to carefully and quantitatively validate their image analysis pipeline. Additionally, DL models should also be carefully validated, and their use (including the training datasets) should be reported appropriately in publications (see Refs. [9,90]).

## Future perspectives

The last few years have seen an explosion in image analysis software, greatly empowering live cell imaging acquisition and analysis. However, tools specifically designed for video analysis, which capitalize on the temporal coherence of live microscopy datasets, have been comparatively scarce. We expect the future will bring software that fully harnesses the dynamic dimension of microscopy videos.

We are especially excited about ongoing developments, including the rise of large segmentation models, such as Segment-Anything [91] and Track-Anything [92], which will facilitate the analysis of microscopy videos. In addition, large language models, such as ChatGPT or Github Copilot, reshape how we develop image analysis pipelines. An exciting development in this context is using natural language to control image analysis software directly, as demonstrated by the Napari plugin Omega [13,93]. These technological strides hint at a not-too-distant future where integrating these tools with self-driving microscopy software will create more interactive and user-friendly self-driving microscopes.

## Data Availability

Data will be made available on request.
